# The influence of organic matter content and media compaction on the dispersal of entomopathogenic nematodes with different foraging strategies

**DOI:** 10.1017/S0031182017001317

**Published:** 2017-08-14

**Authors:** APOSTOLOS KAPRANAS, ABIGAIL M. D. MAHER, CHRISTINE T. GRIFFIN

**Affiliations:** Department of Biology, Maynooth University, Maynooth, Co. Kildare, Ireland

**Keywords:** entomopathogenic nematodes, soil organic matter, peat, soil compaction, foraging behaviour, dispersal, infection, sex ratio

## Abstract

In laboratory experiments, we investigated how media with varying ratio of peat:sand and two levels of compaction influence dispersal success of entomopathogenic nematode (EPN) species with different foraging strategies: *Steinernema carpocapsae* (ambusher), *Heterorhabditis downesi* (cruiser) and *Steinernema feltiae* (intermediate). Success was measured by the numbers of nematodes moving through a 4 cm column and invading a wax moth larva. We found that both compaction and increasing peat content generally decreased EPN infective juvenile (IJ) success for all three species. Of the three species, *H. downesi* was the least affected by peat content, and *S. carpocapsae* was the most adversely influenced by compaction. In addition, sex ratios of the invading IJs of the two *Steinernema* species were differentially influenced by peat content, and in the case of *S. feltiae*, sex ratio was also affected by compaction. This indicates that dispersal of male and female IJs is differentially affected by soil parameters and that this differentiation is species-specific. In conclusion, our study shows that organic matter: sand ratio and soil compaction have a marked influence on EPN foraging behaviour with implications for harnessing them as biological pest control agents.

## INTRODUCTION

Soil is a complex biomaterial (O'Donnell *et al.* 2007) that serves as a reservoir of a wide range of insect pathogens and parasites, such as viruses, bacteria, protozoa, fungi and nematodes (Klingen and Haukeland, 2006). Some of these parasites have soil-transmitted infective stages that need to find hosts, and thus, soil properties modulate biotic interactions between these parasites and their hosts. Soil properties affect patterns of invertebrate locomotion, foraging behaviour and resource exploitation (Kaczmarek, 1978; MacMillan *et al.* 2009; Cornelius and Osbrink, 2010; Kaspi *et al.* 2010; Mathieu *et al.* 2010). Moreover, soil modification, such as compaction (the increase in bulk density or decrease in porosity of soil due to externally or internally applied loads), can have an important influence on soil biota (Aritajat *et al.* 1977; Bouwman and Arts, 2000; Eaton *et al.* 2004; Beylich *et al.* 2010).

Entomopathogenic nematodes (EPN) are one of the most important insect pathogens in soil recognized for their ecosystem services; they have generally broad host range and are commercially produced and marketed around the world as bioinsecticides (Lacey *et al.* 2015). Transmission is achieved by means of a specialized juvenile stage, the infective juvenile (IJ). Typically EPN IJs are applied at the soil surface (e.g. by drenching) with the aim of killing pests feeding in the rhizosphere, such as white grubs (Coleoptera: Scarabaeidae) and weevils (Coleoptera: Curculionidae) (Lacey and Georgis, 2012). An important feature of EPN biology that has implication for their use in biological control is their foraging strategy, that is described as lying on a continuum from ‘ambushing’, sit and wait species to ‘cruising’, more actively moving species (Grewal *et al.* 1994; Campbell *et al.* 2003; Lewis *et al.* 2006). EPN that have been categorized as cruising foragers, such as *Heterorhabditis* and some *Steinernema* species, are allegedly better suited to control soil-dwelling insect pests, whereas nematodes that are categorized as ambush foragers, such as *Steinernema carpocapsae* (Weiser), are traditionally recommended for use against soil surface active pests (Campbell and Gaugler, 1993; Kaya and Gaugler, 1993). However, more recently, it has become clear that *S. carpocapsae* may also be effective against distant, immobile hosts (Dillon *et al.* 2006; Martinez de Altube *et al.* 2008).

A large number of studies have investigated how soil properties, including texture, moisture content and chemistry, affect EPN dispersal and host-finding behaviour and consequently their success in providing control of soil-dwelling insect pests (Stuart *et al.* 2015). Nematodes move in films of moisture over soil particles and pass through the narrow channels between them. What determines the ease with which they move through a soil is largely a function of pore space in relation to the size of the animals themselves (Wallace, 1968). Pore space is a function both of the size of particles (soil texture) and their packing (compaction). Most studies on soil texture have focussed on the mineral components of standard agricultural soils, investigating how the relative proportions of sand, clay and silt influence EPN locomotion and foraging efficiency; these have provided strong evidence that in heavy clay soils, IJ movement is hampered and EPN are not as effective, whereas increasing the proportion of sand improves their success (Georgis and Poinar, 1983; Kung *et al.* 1990; Portillo-Aguilar *et al.* 1999; Koppenhöfer and Fuzy, 2006; Kaspi *et al.* 2010; El-Borai *et al.* 2012). Bulk density (compaction) interacts with soil texture to influence pore size and hence the locomotion of soil inhabitants (Hunt *et al.* 2001), and has been shown to affect dispersal and infection by EPN in a species-specific manner (Portillo-Aguilar *et al.* 1999; Gruner *et al*. 2007), though this factor has received rather little attention. Similarly, little attention has been paid to the effect that the organic matter content of soils may have on EPN efficacy. The limited number of studies that have been done have compared soil types that also differ in properties other than organic matter content, e.g. comparing highly organic soil or potting mix against mineral soils with low organic matter (Choo and Kaya, 1991; Koppenhöfer and Fuzy, 2006), or correlations across field soils varying in organic matter content (Kaspi *et al.* 2010). These studies provide evidence that organic matter can have an important influence on EPN behaviour and efficacy, with differences between EPN species as to which conditions are best. A unique (to our knowledge) study investigated the effect of amending organic media with sand. Addition of up to 50% sand by volume improved the foraging success of *Steinernema riobrave* (Cabanillas, Poinar and Raulston), but not of *Heterorhabditis bacteriophora* (Poinar) (Nielsen and Lewis, 2011). This type of study is of relevance in designing the ideal potting mix for EPN, which have been shown to be effective in a range of organic soil-less media used in nurseries (Oetting and Latimer, 1991; Ansari and Butt, 2011; Nielsen and Lewis, 2011), but also in addressing more fundamental questions regarding foraging type and niche specialization.

Recently, it has been suggested that the ability of nematodes with ambush foraging strategies, such as *S. carpocapsae*, to move efficiently in organic media, such as peat, and parasitize soil pests there, indicates that they are habitat specialists rather than having different foraging strategies (Kruitbos *et al*. 2010; Wilson *et al*. 2012). It is claimed that this holds true because in numerous field studies, *S. carpocapsae* appears to be effective against immobile pests either within soils, some of them characterized by high content of organic matter, such as peat (Dillon *et al.* 2006, 2007; Torr *et al*. 2005; Martinez de Altube *et al.* 2008; Dembilio *et al*. 2010; Williams *et al.* 2013; Kapranas *et al.* 2017*a*, *b*), or within other media, such as wood and leaf litter (Lacey and Unruh, 1998). In addition, it has been suggested that in steinernematid nematodes, and depending on the species, either males of females have superior capabilities in colonizing hosts (Grewal *et al.* 1993; Bohan and Hominick, 1997). In this study, we use laboratory assays to address how EPN species with different foraging strategies move downward in media of varying organic matter (peat) and compaction. Moreover, we explore whether any of these species can be characterized as better adapted to disperse in organic matter, as shown for *S. carpocapsae*, examining thus evidence for the habitat specialization hypothesis (Kruitbos *et al*. 2010; Wilson *et al*. 2012). We also hypothesize that, if indeed there are differences between female and males in their ability to colonize hosts, then we predict that there will be sex ratio shifts in the infecting IJs as the conditions for their dispersal become more or less favourable.

## MATERIALS AND METHODS

### Nematodes

The nematodes used in this experiment were: *Heterorhabditis downesi* Stock, Griffin & Burnell K122, *Steinernema feltiae* (Filipjev) 4CFMO and *S. carpocapsae*. All nematodes were cultured at 20 °C in late instar larvae of the greater wax moth, *Galleria mellonella* (L.) (obtained from The Mealworm Company, Sheffield, UK), using standard protocols (Kaya and Stock, 1997). Harvested IJs were washed three times by sedimentation in tap water and stored at 9 °C until use.

### Assay

A single *G. mellonella* larva was enclosed in a wire cage and placed in the bottom of a 60 cm^3^ plastic pot (4 cm × 4·4 cm, H × D, with snap on lid; Wains of Tunbridge Wells, UK) and peat/sand mix was added to each pot. Peat (Shamrock Irish Moss Peat, Bord na Mona Horticulture, Newbridge, Ireland) was passed through a 1 cm sieve, and visible pieces of plant material were removed by hand. Sand (B & Q Play Pit sand, Kingfisher plc, London, UK) was passed through a 1·4 mm sieve. Moisture content of sand was adjusted to 8% (w/w). The moisture content of the peat was on average 57% (ranging from 54 to 60·7%). Five media were prepared, with the following ratios of peat:sand (v/v): 100:0, 75:25, 50:50, 25:75 and 0:100. For non-compacted treatments, pots were filled to the top with peat:sand mixture, which was levelled off. For compacted treatments, soil was added to the pot, compacted by tapping the pot three times on a flat surface and applying light pressure with the thumbs. More mixture was then added, compacted by applying light pressure with the thumbs and levelled off at the top. A small depression was made in the centre of each pot and 100 IJs in 100 *µ*L of tap water were added. The filled weight of pots was recorded (Table 1). Pots were lidded and incubated at 20 °C for 24 h. Insects were removed from the soil and incubated at 20 °C for a further 3 days to allow them to die and the IJs inside to develop to adult. Any insects that showed the characteristic colour and appearance of nematode infection were dissected and the number of first-generation adult nematodes was counted. For steinernematids, the sex of the nematodes was recorded. Both live and dead adults were counted, as steinernamatid males might appear dead due to fatal fights, but can be observed easily upon dissection of the *Galleria* larvae (O'Callaghan *et al.* 2014; Zenner *et al.* 2014; Kapranas *et al.* 2016). In a small number of insects displaying the characteristics of nematode infection, no nematodes were found and these were recorded as a single nematode invasion. Any insects with signs of death – other than those caused by EPN (distinguished by characteristic colour and consistency) – were excluded from the dataset.

**Table 1.** Bulk density (g/100 cm^3^) of peat–sand mixture in different treatments of sand content and compaction^a^

	Uncompacted		Compacted			
Sand (%)	Average ± s.e.	Range	Average ± s.e.	Range	*t*^b^	*P*
0	16·49 ± 0·069a	14·24–17·53	23·15 ± 0·102a	21·23–24·85	53·81	<0·001
25	25·79 ± 0·138b	22·41–29·20	37·24 ± 0·183b	33·48–41·9	49·77	<0·001
50	34·81 ± 0·200c	31·76–40·19	48·77 ± 0·243c	45·45–54·55	44·25	<0·001
75	42·21 ± 0·2046d	37·22–46·28	57·62 ± 0·233d	52·38–62·58	49·60	<0·001
100	52·99 ± 0·313e	49·01–59·28	65·14 ± 0·1848e	60·76–69·33	33·35	<0·001
*F*_4,384_	4896·52		7186·47			
*P*	<0·001		<0·001			

a Different letters show significant differences within column (one-way analysis of variance followed by a *post hoc* Tukey–Kramer test, *α* = 0·05).

b One-tailed *t*-test (uncompacted < compacted), across rows.

There were four or five rounds of the experiment for each nematode species. Each round of the experiment consisted of five or eight replicate pots for each of 10 treatments (five levels of peat:sand mixture and two levels of compaction) for one nematode species. Each round was conducted with a different culture batch of nematodes. The total number of replicate pots per treatment was 23, 26 or 28 for *S. carpocapsae*, *H. downesi* and *S. feltiae*, respectively. Controls (no nematodes) were not included as in our experience natural mortality of wax moths is negligible in these assay conditions.

### Statistical analysis

Generalized linear modelling was used to explore the influence of EPN species, sand content and compaction on mortality rates of *Galleria* larvae and also on the numbers of nematode IJs entering the host. We assumed binomial error variance for mortality data and quasi-Poisson error variance for IJ numbers infecting, which in most cases were small integer data, using where possible empirically estimated scale parameters to account for potential overdispersion. After rescaling, the significance of explanatory variables was assessed by *F* ratio tests and by the change in deviance when a variable was removed from the full model. The influence of sand content and compaction on sex ratio of invading IJs (proportion males) data were explored with logistic regression assuming quasi-binomially distributed errors to counter the effects of overdispersion (Wilson and Hardy, 2002). Since multiple culture batches for each EPN species were used, we also ran generalized linear mixed models with each culture batch fitted as a random variable to confirm the result of the initial analyses, but presented only if results are different. Further sub-analyses for each species were run separately to explore possible significant interactions between species and sand content and also between species and compaction. All analyses were performed in Genstat V14.1 (VSN International, Hemel Hempstead, UK).

## RESULTS

### Mortality of *Galleria* larvae

**Fig. 1.**
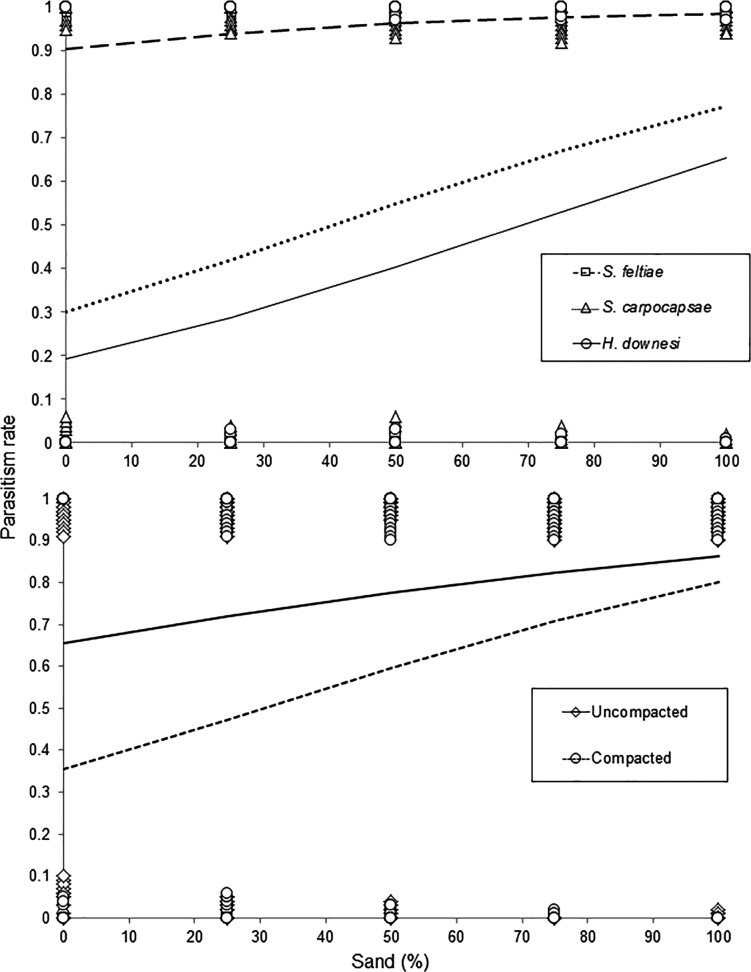
The influence of sand content (A) and media compaction (B) on the parasitism rates of wax moth larvae caused by three species of entomopathogenic nematodes (logistic regression).

**Fig. 2.**
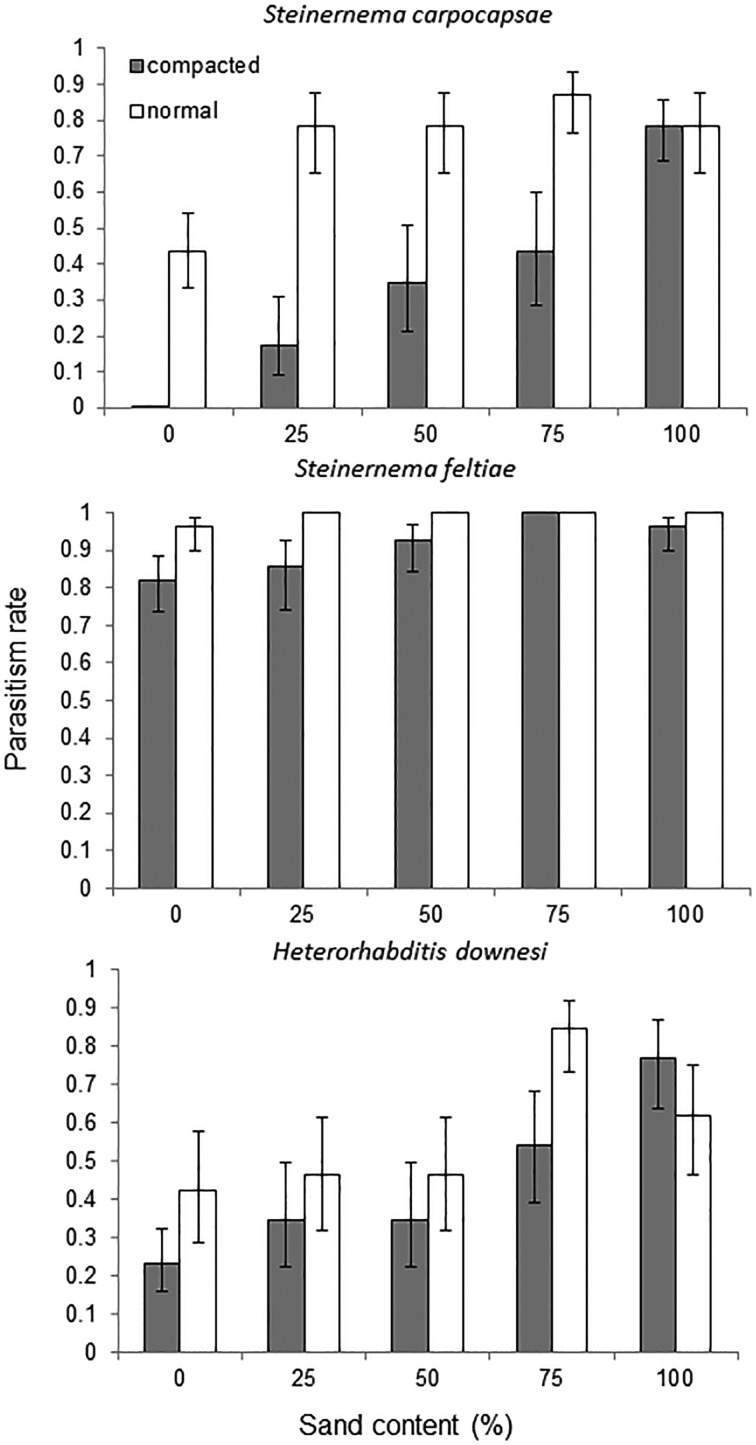
Parasitism rates of wax moth larvae by *Steinernema carpocapsae*, *Heterorhabditis downesi* and *Steinernema feltiae* in media varying in peat:sand content and level of compaction.

The mortality (= parasitism) of *Galleria* larvae was on average 53·9, 95·3 and 50·1% for *S. carpocapsae*, *S. feltiae* and *H. downesi*, respectively. The mortality of *Galleria* larvae was influenced by EPN species (*F*_2,762_ = 92·36, *P* < 0·001, Fig. 1A), was positively correlated with sand content (*F*_1,762_ = 66·62, *P* < 0·001, Fig. 1A and B) but was adversely affected by compaction (*F*_1,762_ = 36·94, *P* < 0·001, Fig. 1B). There were also significant interactions between species and compaction (*F*_2,762_ = 6·87, *P* = 0·001) and between sand content and compaction (*F*_2,762_ = 5·81, *P* = 0·016). For *S. carpocapsae*, both sand content (*F*_4,220_ = 7·56, *P* < 0·05) and compaction were significant (*F*_1,220_ = 22·52, *P* < 0·001, Fig. 2). For *H. downesi*, sand content was significant (*F*_4,250_ = 6·50, *P* < 0·001), whereas compaction was marginally not significant (*F*_1,250_ = 3·63, *P* = 0·058, Fig. 2). Lastly for *S. feltiae*, where mortality was close to 100% across treatments, neither sand content (*F*_1,274_ = 0·62, *P* = 0·648, Fig. 2) nor compaction was significant (*F*_1,274_ = 0·62, *P* = 0·107, Fig. 2).

### Number of IJs infecting *Galleria* larvae

**Fig. 3.**
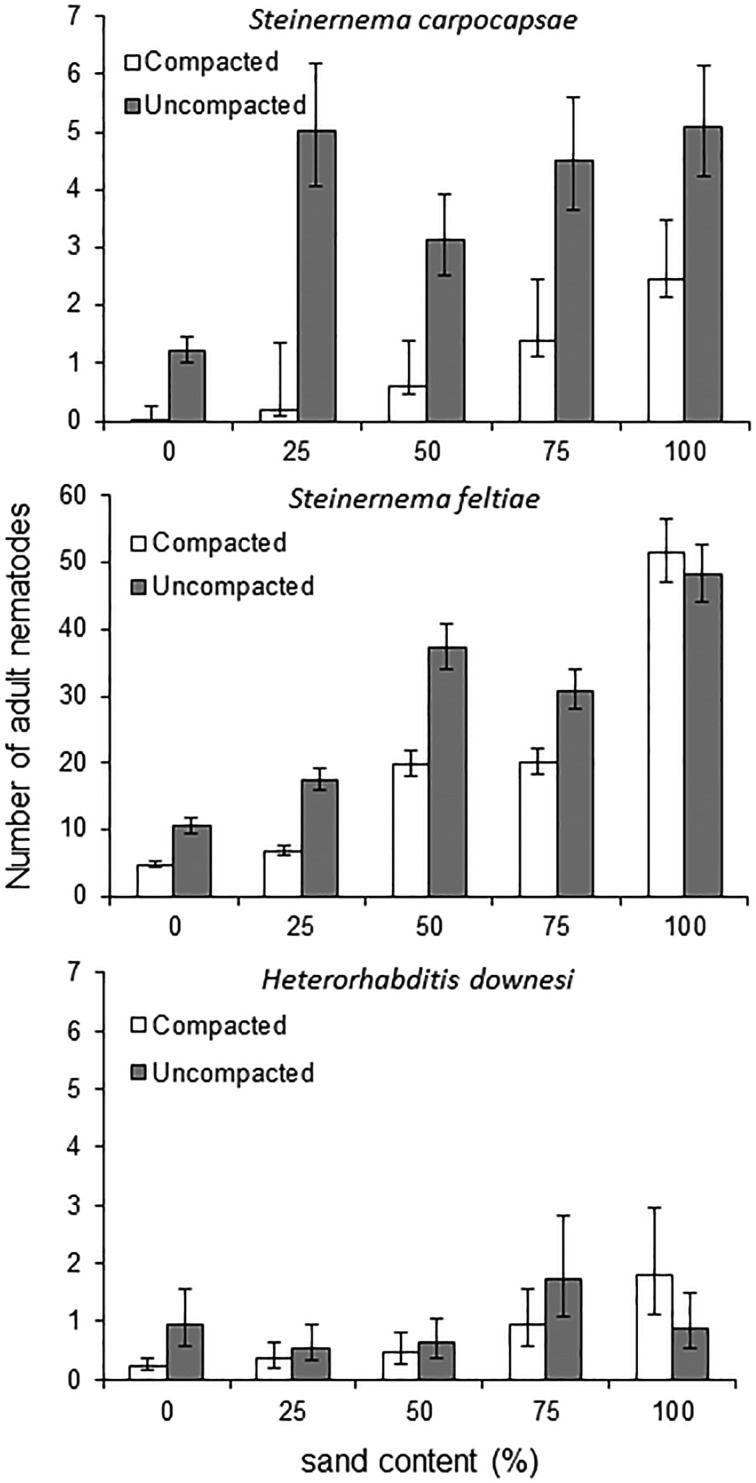
Number of adult nematodes of *Steinernema carpocapsae*, *Heterorhabditis downesi* and *Steinernema feltiae* found upon dissection of wax moth larvae subjected to nematode infection in media varying in peat:sand content and level of compaction.

The numbers of IJs infecting *Galleria* larvae were influenced by all factors introduced in the model; in addition, second-level interactions between main effects were significant (Table 2). In summary, higher sand content led to higher number of IJs infecting the *Galleria* larvae, whereas compaction led to fewer IJs infecting the *Galleria* larvae (Fig. 3). *Galleria* larvae infected by *S. feltiae* had a greater number of nematodes (up to 122) upon dissection than *Galleria* larvae infected by *S. carpocapsae* and *H. downesi*, where the maximum numbers were 22 and 10, respectively (Fig. 3). Sub-analysis for each species separately revealed that in every case, sand content, compaction and their interaction were significant (data not shown).

**Table 2.** Log-linear analysis of factors influencing the number of nematode IJs infecting *Galleria* larvae

Effect	d.f.	Deviance	*F*	*P*
Nematode species	2	9915·772	697·71	<0·001
Sand	1	2351·493	330·92	<0·001
Compaction	1	296·133	41·67	<0·001
Nematode species×sand	2	20·345	1·43	0·240
Nematode species×compaction	2	113·981	8·02	<0·001
Sand×compaction	1	240·001	33·77	<0·001
Nematode species×sand×compaction	2	15·920	1·12	0·327
Residual	758	5386·332		
Total	769	18 339·977		

### Medium and sex ratios in steinernematid nematodes

**Fig. 4.**
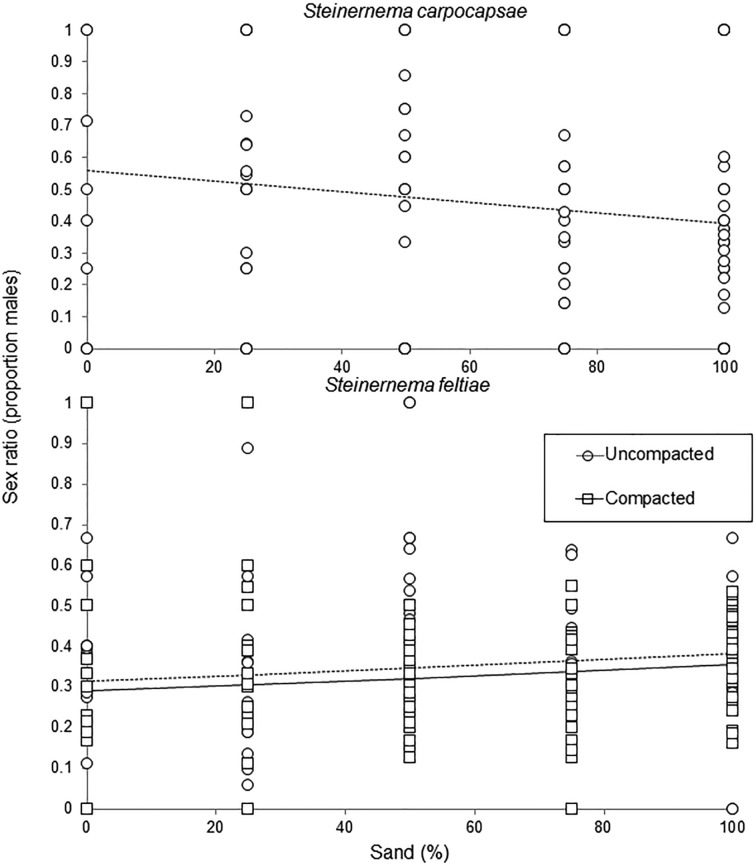
The influence of peat:sand content and media compaction on sex ratios (proportion males) of *Steinernema carpocapsae* and *Steinernema feltiae* (logistic regression). Compaction is not significant for *Steinernema carpocapsae* and regression lines are presented only for the influence of sand content.

*Steinernema carpocapsae* adult sex ratios upon cadaver dissection were not influenced by the number of individual adults (*F*_1,123_ = 0·56, *P* = 0·456) or compaction (*F*_1,123_ = 0·39, *P* = 0·533), but sex ratios significantly decreased with increasing sand content (*F*_1,123_ = 4·47, *P* = 0·037, Fig. 4). Sex ratios of about 55·7% males in pure peat shifted to an average 39·4% males in pure sand as suggested by our regression model. Similarly, *S. feltiae* adult sex ratios were not influenced by the number of individual adults (*F*_1,259_ = 3·38, *P* = 0·067) and were marginally decreased in compacted media (*F*_1,259_ = 3·25, *P* = 0·072, but a mixed model indicated that compaction was significant: *F*_1,259_ = 3·99, *P* = 0·047). Moreover, *S. feltiae* sex ratios significantly increased with increased sand content (*F*_1,259_ = 7·08, *P* = 0·008, Fig. 4). Thus, sex ratios ranged from 29·7% males in pure peat to 35·5% males in pure sand for compacted media, and from 31·5 to 38·2% males (peat to sand) in uncompacted media.

## DISCUSSION

Our experiments assessed the ability of three EPN species representing different foraging strategies to move through (and infect insects in) media with various peat:sand ratios and two levels of compaction, and showed that both factors had a marked influence, although these effects need to be addressed on an individual species level. A high sand content of soils generally facilitates nematode movement, though for the most part comparisons are done on soils with varying proportions of sand, silt and clay and rather low (<5%) organic matter content (Georgis and Poinar, 1983; Kung *et al.* 1990; Portillo-Aguilar *et al.* 1999; Kaspi *et al.* 2010; El-Borai *et al.* 2012). In our study, increasing the sand content of the peat:sand mixture resulted in increasing numbers of nematodes finding and invading wax moth larvae for each of the three species tested: *H. downesi* (a cruise forager), *S. carpocapsae* (an ambusher) and *S. feltiae*, a species sharing properties of both foraging strategies (Campbell *et al.* 2003; Campos-Herrera and Gutiérrez, 2014). The trend was less evident for *S. carpocapsae* and *H. downesi* in uncompacted media. Similarly, physically altering various organic media with sand (up to 50% volume) improved foraging distance for *S. riobrave*, but not for *H. bacteriophora* (Nielsen and Lewis, 2011).

It is noteworthy that for *S. carpocapsae*, the lowest number of nematodes invading occurred in pure peat for both levels of compaction, much lower than in pure sand. This is in contrast to the findings of Kruitbos *et al*. (2010) who reported that *S. carpocapsae* did not display host finding in pure sand, but did in pure peat. It is notoriously difficult to compare across studies where details of assays, materials and nematode strains differ. For example, Kruitbos *et al*. (2010) measured relative proportions of nematodes moving towards or away from a host (and did not report absolute numbers of nematodes moving), while we measured numbers reaching (and invading) a host, where arrival at the host through the medium could have resulted either from host-specific orientation or random dispersal of the IJs. Thus, while it may be that orientation of *S. carpocapsae* towards a distant host is facilitated in peat, as shown by Kruitbos *et al*. (2010), our findings indicate that movement as measured by parameters more closely aligned with fitness (host death and nematode establishment) is impeded in pure peat relative to all other mixtures tested. Thus, our results do not support the hypothesis that *S. carpocapsae* is an organic habitat specialist. No mixture containing peat yielded a higher proportion of *S. carpocapsae* invading wax moths than pure sand, and in compacted media increasing the proportion of peat resulted in a steady decrease in invasion rate. While it is clear that *S. carpocapsae* can find and infect distant hosts, including in organic media (Lacey and Unruh, 1998; Dillon *et al.* 2006, 2007; Torr *et al*. 2005; Martinez de Altube *et al.* 2008; Dembilio *et al*. 2010; Santhi *et al.* 2015; Kapranas *et al.* 2017*a*), there is insufficient field data to indicate habitat specialization. An alternative hypothesis is that there are some IJs with enhanced dispersal ability relative to the average dispersal ability of the whole population and recent studies successfully selecting for better dispersing *S. carpocapsae* add some weight to this argument (Bal *et al.* 2014*a*,*b*; Santhi *et al.* 2016). A potential criticism of the experiment is that when peat and sand are mixed to create the different media, the moisture level may also change, and consequently the observed responses of our experiments may be influenced by differences in humidity as well as sand content. However, the moisture content in both pure sand and pure peat was optimal for nematode movement, and so it is unlikely that nematode movement was seriously impacted by sub-optimal moisture in any mixture. Therefore, the differences detected in the dispersal assays were due to the intended treatments (compaction, sand:peat ratios) rather than being an artefact of differing available moisture contents. A similar approach was employed also by Nielsen and Lewis (2011) who used dry sand to prepare a dilution series of organic media. While all three nematode species were negatively impacted by compaction of the medium, the extent to which they were affected differed between species. Soil compaction had a more adverse effect on *S. carpocapsae* than on the other two species, while *H. downesi* was the least affected. A similar differential response to increased bulk density (compaction) of mineral soils was reported by Portillo-Aguilar *et al.* (1999), with *S. carpocapsae* being more affected than either *H. bacteriophora* or *S. glaseri* (both of which are cruise foragers). For species such as S. *carpocapsae* that nictate (Campbell and Gaugler, 1993; Campbell and Kaya, 1999), lifting their body off the substrate can aid in bridging pores in soils and in organic media (Croll, 1970; Wilson *et al*. 2012) as well as being a strategy for attaching to passing hosts. Thus, compaction might have diminished their ability to move by bridging between particles within such media. Sand cannot be compacted as much as peat, so increasing sand content could improve properties of compacted soil. In the present study, the increase in sand ameliorated the negative effects of compaction, though *S. carpocapsae* was still negatively impacted by compaction of pure sand.

It is evident that higher IJ invasion correlates with higher insect mortality in our experiments. *Steinernema feltiae* IJs were the most successful in invading and consequently killing most of the *Galleria* larvae in a broad range of media varying in peat content and compaction, even at the most adverse conditions of low sand content. For both *S. carpocapsae* and *H. downesi*, only a small proportion of IJs (typically 1–5 of the 100 applied) infected *Galleria* hosts in media of any kind (% peat-sand and compaction), compared with up to 50 IJs in *S. feltiae*.

Examination of the sex ratios of the adult steinernematid nematodes within infected wax moths allows us to make inferences regarding the sex ratios of the infecting IJs. The composition of the medium affected the sex ratio of both species, but in opposite directions. As the proportion of sand increased, the proportion of males decreased for *S. carpocapsae*, and increased for *S. feltiae*. Alsaiyah *et al.* (2009) found that *S. carpocapsae* sex ratios in a similar sand assay did not differ from the sex ratio of nematodes developing in haemolymph (representing the underlying population sex ratio without influence of migration or responses to host). Thus, we can assume that in pure sand, male and female IJs of *S. carpocapsae* dispersed and invaded at similar rates (the sex ratio of invaders did not deviate from the population sex ratio of 0·43 as assessed *in vitro*). The results of the present study indicate that an increasing proportion of peat in the medium increasingly favours host finding and invasion by male *S. carpocapsae*, resulting in a deviation from the underlying population sex ratio, which becomes male-biased at higher peat contents. For *S. feltiae*, on the other hand, Alsaiyah *et al.* (2009) found that females in the population are more likely to invade than males (proportion males 0·35 in insects in sand assay compared with 0·43 in haemolymph). From the present study, there is evidence that this deviation from the population sex ratio of *S. feltiae* increases further in favour of females as the proportion of peat in the medium increases and under conditions of compaction. Similarly, Campos-Herrera and Gutiérrez (2014) found that the proportion of *S. feltiae* females in *G. mellonella* was higher in an assay requiring the IJs to migrate (conditions that restricted the number of nematodes entering the insects), than in a close-contact penetration assay. Previous studies on steinernematids suggest that differences between male and female IJs can have consequences for host colonization (Grewal *et al.* 1993; Bohan and Hominick, 1997; Alsaiyah *et al.* 2009). In our experiments, we show that in conditions hampering nematode movement (lower sand content and compacted medium), there are small but significant sex ratio shifts; this is direct evidence that there are sex-specific differences in dispersal of IJs, in a species-specific manner. Further research is warranted into the proximate mechanisms by which the physical structure of the medium affects sex ratio of IJs finding and invading hosts, and the adaptive significance of the phenomenon.

In conclusion, both peat content and compaction influence nematode movement and efficacy. Our results suggest that nematodes, irrespective of their foraging strategy, move better through the soil matrix as the sand content increases, with the best performing species being *S. feltiae*, a species with intermediate foraging behaviour. We also show that the ratio of peat:sand can differentially influence the foraging behaviour of female and male IJs indicating that IJ infection patterns depend on interactions between their sex and physical properties of the medium in which they disperse.
